# Requirement of DLG1 for Cardiovascular Development and Tissue Elongation during Cochlear, Enteric, and Skeletal Development: Possible Role in Convergent Extension

**DOI:** 10.1371/journal.pone.0123965

**Published:** 2015-04-10

**Authors:** Akiko Iizuka-Kogo, Takao Senda, Tetsu Akiyama, Atsushi Shimomura, Ryuji Nomura, Yoshimi Hasegawa, Ken-ichi Yamamura, Hiroshi Kogo, Nobuhiko Sawai, Toshiyuki Matsuzaki

**Affiliations:** 1 Department of Anatomy I, Fujita Health University School of Medicine, Aichi, Japan; 2 Department of Anatomy and Cell Biology, Gunma University Graduate School of Medicine, Gunma, Japan; 3 Department of Anatomy, Gifu University Graduate School of Medicine, Gifu, Japan; 4 Laboratory of Molecular and Genetic Information, Institute of Molecular and Cellular Biosciences, The University of Tokyo, Tokyo, Japan; 5 The Department of Communication Disorders, School of Psychological Science, Health Sciences University of Hokkaido, Hokkaido, Japan; 6 Division of Developmental Genetics, Institute of Resource Development Analysis, Kumamoto University, Kumamoto, Japan; University of Queensland, AUSTRALIA

## Abstract

The *Dlg1* gene encodes a member of the MAGUK protein family involved in the polarization of epithelial cells. Null mutant mice for the *Dlg1* gene (*Dlg1^-/-^* mice) exhibit respiratory failure and cyanosis, and die soon after birth. However, the cause of this neonatal lethality has not been determined. In the present study, we further examined *Dlg1^-/-^* mice and found severe defects in the cardiovascular system, including ventricular septal defect, persistent truncus arteriosus, and double outlet right ventricle, which would cause the neonatal lethality. These cardiovascular phenotypes resemble those of mutant mice lacking planar cell polarity (PCP) genes and support a recent notion that DLG1 is involved in the PCP pathway. We assessed the degree of involvement of DLG1 in the development of other organs, as the cochlea, intestine, and skeleton, in which PCP signaling has been suggested to play a role. In the organ of Corti, tissue elongation was inhibited accompanied by disorganized arrangement of the hair cell rows, while the orientation of the stereocilia bundle was normal. In the sternum, cleft sternum, abnormal calcification pattern of cartilage, and disorganization of chondrocytes were observed. Furthermore, shortening of the intestine, sternum, and long bones of the limbs was observed. These phenotypes of *Dlg1^-/-^* mice involving cellular disorganization and insufficient tissue elongation strongly suggest a defect in the convergent extension movements in these mice. Thus, our present results provide a possibility that DLG1 is particularly required for convergent extension among PCP signaling-dependent processes.

## Introduction

In multicellular organisms, two subcellular compartments in single cells often become differently specialized in structure and function according to the tissue functions. This organization of subcellular components and structures is known as cellular polarization [1]. In epithelial tissues covering the surface of organs, two kinds of polarization are observed, namely apicobasal polarity and planar cell polarity (PCP). Apicobasal polarity is formed between the two distinct plasma membrane compartments of the basal and apical cell membranes. Conversely, the polarity orthogonal to the apicobasal axis is called PCP and determines the orientation of the cells within the horizontal plane. For example, PCP is reflected in the asymmetric positioning and coordinated rotation of motile cilia in the embryonic node [2] and the orientation of the V-shaped stereocilia on the apical surface of hair cells in the organ of Corti [3]. During PCP formation in *Drosophila* for example, the PCP signaling pathway including core PCP components, composed of DSH, FZ, VANG, STAN, PK, and DGO, plays a central role [1]. The PCP signaling pathway is required not only for the above-mentioned planar cell polarization, but also for dynamic tissue movement during organogenesis [4]. This tissue movement is called convergent extension (CE). In CE, cells within a tissue sheet intercalate with each other to form a tissue that is narrow in width and long in longitudinal axis [5]. Mutant mice lacking functional PCP components exhibit characteristic phenotypes, including failure of neural tube closure, open eyelids, misorientation of the stereocilia of cochlear hair cells, and malformation of the outflow tract in the cardiovascular system [3, 6, 7]. However, the extents of the dependence of these phenotypes on PCP or CE remain to be elucidated.

In cardiovascular development, the outflow tract is originally formed as a single tube connecting to the right ventricle and then changes its position leftward. Simultaneously, conotruncal cushions develop within the outflow tract and fuse to form the conotruncal septum separating the aorta and the pulmonary artery. In addition, conotruncal cushions fuse to endocardial cushions to close the ventricular septum and to separate the pulmonary and systemic circulations [8]. Two progenitor cell lineages are known to be critical for the development of the outflow tract. Specifically, a secondary heart field (SHF), comprising a splanchnic mesoderm caudal to the outflow tract, contributes to cardiomyocytes of the outflow tract [9]. The other cell lineage is composed of cardiac neural crest cells, which migrate into the outflow tract and form conotruncal cushions [10]. Abnormal behaviors of these cell types can cause congenital heart defects. For example, neural crest-specific gene targeting of ACVR1/ALK2 impaired migration of these cells, disturbed the separation of the outflow tract, and caused persistent truncus arteriosus (PTA) [11]. As another example, impaired development of the SHF caused malposition of the pulmonary and aortic arteries and resulted in double outlet right ventricle (DORV) [12–14]. Furthermore, PCP signaling is thought to be necessary for development of the outflow tract, because mutant mice for PCP component genes such as *Vangl2*, *Wnt5a*, *Scrib*, *Fzd*, and *Dvl* exhibit cardiovascular defects including PTA and DORV [6, 15–20].

*Drosophila* Discs large (DLG) is a membrane-associated guanylate kinase (MAGUK) protein with three PDZ domains and a guanylate kinase-like domain, and has been identified as a tumor suppressor involved in apicobasal polarization of epithelial cells [21, 22]. In epithelial cells, DLG coordinates with LGL and SCRIB and localizes PAR3/PAR6/aPKC complexes at the apical membrane to form apicobasal polarity [23]. During asymmetric cell division of neural stem cells and sensory organ precursor cells, DLG is involved in the spindle orientation [24, 25]. DLG1 is one of the four mammalian homologs of *Drosophila* DLG protein and forms the MAGUK scaffold protein family along with three other DLG homologs, DLG2/CHAPSYN-110, DLG3/SAP102, and DLG4/PSD95. DLG1 is widely expressed in various cell types and accumulates at cell-cell contact sites in polarized epithelial cells [26–28].

To date, several *Dlg1* gene-targeted mice have been generated by us and other researchers, and the developmental roles of DLG1 have been analyzed [26, 28–30]. These studies showed that loss of DLG1 function leads to complete neonatal lethality [26, 28, 30]. In addition, respiratory failure [28, 30], cyanosis [26, 28], and defects in craniofacial [26, 28] and urogenital [28–30] organs have been reported. Because DLG is involved in apicobasal polarization in the *Drosophila* epithelium, we had expected that the essential function of DLG1 protein would also be the formation of apicobasal polarity in mice, and that the loss of apicobasal polarity would cause the developmental defects observed in *Dlg1* ^*-/-*^ mice. In our previous study, however, we found that the apicobasal polarity appeared to be maintained in the absence of DLG1, as the intracellular localizations of CDH1/E-cadherin and TJP1/ZO-1 in ureteric epithelial cells were normal in *Dlg1* ^*-/-*^ mice [28]. Consequently, the cell biological function of DLG1 during mouse development has not been clarified. Therefore, in the present study, we further analyzed *Dlg1* ^*-/-*^ embryonic mice to identify the developmental problems causing the neonatal lethality and to understand the underlying functional mechanism of DLG1. Here we demonstrate the novel requirement of DLG1 for the development of the cardiovascular system and for tissue elongation during cochlear, enteric, and skeletal development, and propose that DLG1 is likely involved in the CE process.

## Materials and Methods

### Animal experiments and ethics statement

*Dlg1* ^*-/-*^ mice were maintained by brother-sister mating on a mixed genetic background derived from C57BL/6N and CBA/J strains [28]. Embryos obtained from timed mating of heterozygous *Dlg1* gene-targeted (*Dlg1* ^*+/-*^) mice were used for experiments. In addition, *Dlg1* ^*-/-*^ mice whose genetic background was purified to C57BL/6J by backcrossing over 10 times were used for analyses of the cardiovascular system and hair cell numbers and for measurements of the length of the organ of Corti. Because *Dlg1* ^*+/-*^ mice exhibit normal development in utero and postnatal viability, both wild-type (*Dlg1* ^*+/+*^) and *Dlg1* ^*+/-*^ mice were used as controls in the present study. C57BL/6J-Tg(P0-Cre)94Imeg and B6;D2-Tg (CAG-CAT-EGFP) 39Miya (kind gift from Dr. J. Miyazaki) transgenic mouse strains were crossed to yield P0-Cre/EGFP transgenic mice for fluorescence visualization of neural crest-derived cells. Embryos and neonatal mice were euthanized by decapitation, and adult mice by careful cervical dislocation by trained personnel. All animal experiments were reviewed and approved by the Institutional Animal Care and Use Committee of Fujita Health University and/or the Institutional Animal Care and Experimentation Committee of Gunma University (approval number 12–051), and were conducted in accordance with the Regulations for the Management of Laboratory Animals at Fujita Health University, the Guidelines for Animal Experiments of Gunma University, Japanese Law (No. 105) and Notification (No. 6) of the Japanese Government, and the Committee on Ethics in Japan.

### Morphological analysis of the cardiovascular system

For morphological analysis of the prenatal heart and vascular system, a total of 29 (17 CTRL and 12 *Dlg1* ^*-/-*^) embryos derived from *Dlg1* ^*+/-*^ parental mice with a mixed background and 15 (10 CTRL and 5 *Dlg1* ^*-/-*^) embryos derived from *Dlg1* ^*+/-*^ parental mice with the C57BL/6J background at embryonic day (E) 18.5 were used. Among these mice, the gross morphology was observed in 24 (15 CTRL and 9 *Dlg1* ^*-/-*^) embryos with the mixed background and all 15 embryos with the C57BL/6J background. For histological observation, 12 (6 CTRL and 6 *Dlg1* ^*-/-*^) embryos with the mixed background and 7 (2 CTRL and 5 *Dlg1* ^*-/-*^) embryos with the C57BL/6J background were used. Histology during development was observed in 8 (5 CTRL and 3 *Dlg1* ^*-/-*^) embryos with the mixed background at E11.5. For the histological observation, the thoracic organs were fixed with 4% paraformaldehyde, cut into sequential paraffin-embedded sections, and stained with hematoxylin and eosin. Development of the outflow tract was also observed using scanning electron microscopy. The heart was dissected out from 15 (10 CTRL and 5 *Dlg1* ^*-/-*^) embryos with the mixed background at E12.5, trimmed to open the lumen of the right ventricle and outflow tract, and prepared using a conventional method [31]. An S-2600 scanning electron microscope (Hitachi, Tokyo, Japan) was used for observation.

For fluorescence visualization of neural crest-derived cells in *Dlg1* ^*-/-*^ mice, P0-Cre and CAG-CAT-EGFP transgenes were introduced into the mice with the C57BL/6J background by crossing [32]. Migration of neural crest-derived cells into the outflow tract was observed in embryos at E9.5 (2 control mice), E10.5 (3 control mice and 3 *Dlg1* ^*-/-*^ mice), and E11.5 (19 control mice and 13 *Dlg1* ^*-/-*^ mice) using a fluorescence dissecting microscope (SteREOLumar.V12; Carl Zeiss, Oberkochen, Germany) equipped with an Axiocam CCD camera.

Immunohistochemical staining for DLG1 protein and smooth muscle actin (SMA) in the heart region was performed as previously described [28]. An anti-DLG1 antibody [33] and anti-SMA antibody (DAKO, Carpinteria, CA, USA) were used.

### Analyses of hair cells in the inner ear

Surface preparation of the organ of Corti was performed for tissue observation, immunohistochemistry for FZD3, and measurement of the cochlear duct [34]. Briefly, the temporal bones were dissected from each embryo and fixed overnight with 4% paraformaldehyde in phosphate-buffered saline (PBS). The cochlear duct was dissected out and its length was measured in 8 control and 4 *Dlg1* ^*-/-*^ intact samples. The surface of the organ of Corti was then exposed and used for staining. The samples were permeabilized with 0.1% Triton X-100 in PBS for 15 min, before staining with phalloidin tagged with Alexa 488 or 568 (Invitrogen, Carlsbad, CA, USA) and DAPI (Dojin, Kumamoto, Japan) overnight at 4°C and thorough washing with 0.01% Triton X-100 in PBS. For FZD3 immunostaining, permeabilized samples were incubated with an anti-FZD3 antibody (GeneTex, Irvine, CA, USA) overnight at 4°C, and then treated with an Alexa-tagged secondary antibody, Alexa-tagged phalloidin, and DAPI. The stained samples were mounted between glass coverslips using ProLongGold (Invitrogen) and observed by fluorescence microscopy (Axiovert; Carl Zeiss) or laser scanning microscopy (LSM710; Carl Zeiss).

The orientation of hair cells in the organ of Corti was observed using 7 control and 6 *Dlg1* ^*-/-*^ embryos at E18.5. The stereocilia of 100 consecutive outer hair cells in the outermost row, lined in the apical direction from a point at 1 mm to the basal end, were observed. The orientation of the stereocilia in each cell was determined by measuring the angle formed by the direction of the hair cell row and a line connecting the basolateral ends of the chevron-shaped stereocilia bundle, and the means of their absolute values were compared between the *Dlg1* ^*-/-*^ and control mice.

The total numbers of hair cells in the organ of Corti were counted in the DAPI- and phalloidin-stained surface preparations of the organ of Corti from 5 control and 4 *Dlg1* ^*-/-*^ embryos at E18.5, for which we could obtain whole intact samples. In these samples, the alignments of supporting cells and hair cells were observed using z-plane reconstruction images from confocal sections.

Cell proliferation was evaluated by 5-bromo-2-deoxyuridine (BrdU) labeling. *Dlg1* ^*+/-*^ pregnant mice received four intraperitoneal injections of BrdU (50 μg/g body weight) between 14.5 and 16.5 days post-coitum. The embryos were collected at E18.5, fixed with 4% paraformaldehyde overnight, and cut into frozen sections of 8-μm thickness to detect BrdU incorporated into the organ of Corti. The sections were autoclaved in target retrieval solution (DAKO) for 15 min at 105°C before incubation with anti-BrdU (Roche, Basel, Switzerland) and anti-myosin 6 (Proteus, Ramona, CA, USA) antibodies. Primary antibodies and nuclei were stained with Alexa-tagged secondary antibodies and DAPI, respectively. The distribution of BrdU-positive cells in the zone of non-proliferative cells [35] was observed and their proportion to the total observed cell number was calculated for inner hair cells, outer hair cells, and Hensen cells. The proportions were averaged over at least three independent experiments. The total numbers of observed cells were 61 and 170 inner hair cells, 155 and 528 outer hair cells, and 188 and 595 Hensen cells in control and *Dlg1* ^*-/-*^ mice, respectively.

### Analysis of bone formation

Mineralization in embryonic bone was visualized by double-staining with Alcian blue and Alizarin red S. Seven embryos were used for each stage and genotype. Embryos at E15.5, E16.5, E17.5, and E18.5 were skinned, eviscerated, and fixed in 100% ethanol. The non-mineralized cartilage was stained with freshly prepared Alcian blue solution containing 0.015% Alcian Blue 8GX (Sigma-Aldrich, St. Louis, MO, USA), 20% acetic acid, and 76% ethanol for 24 h. The samples were briefly washed twice with 95% ethanol and immersed in 2% KOH solution overnight. Mineralized cartilage and bone were then stained with freshly prepared Alizarin red solution containing 0.0075% Alizarin red S (Sigma-Aldrich) and 1% KOH overnight. The specimens were sequentially destained with 1% KOH/50% glycerol solution and 50% glycerol/50% ethanol solution for up to 3 days and observed in 100% glycerol. For histological observation, the sternum at E19.5 was used and paraffin sections were stained with hematoxylin and eosin.

### Morphological analysis of the intestine

For measurement of the gut length in E18.5 embryos, the digestive tract was dissected out from 16 control and 6 *Dlg1* ^*-/-*^ mice, and the length between the pylorus and the distal end of the rectum was measured. For analysis of embryos at an earlier stage, intestines were dissected out from 6 control and 11 *Dlg1* ^*-/-*^ mice, and the enteric nerve plexus was stained by whole-mount immunohistochemistry with an anti-NGFR/p75 antibody (Promega, Fitchburg, WI, USA) and an Alexa 488-conjugated anti-rabbit IgG antibody (Invitrogen). Fluorescence images were captured by SteREOLumar.V12 and AxioCamMRm (Carl Zeiss), and the distances from the pylorus to the distal end of the NGFR/p75 signals, from the pylorus to the ileocecum (length of the small intestine), and from the ileocecum to the distal end of the rectum (length of the large intestine) were measured.

### Statistical analysis

All data were expressed as the mean and standard error. The statistical significance of differences between the mean values in control and *Dlg1* ^*-/-*^ mice was evaluated by an F-test and a *t*-test. All analyses were performed using Microsoft Excel 2010 (Microsoft Corporation, Redmond, WA, USA). Values of p<0.05 were considered to indicate statistical significance.

## Results

### Cardiovascular defects

**Fig 1:**
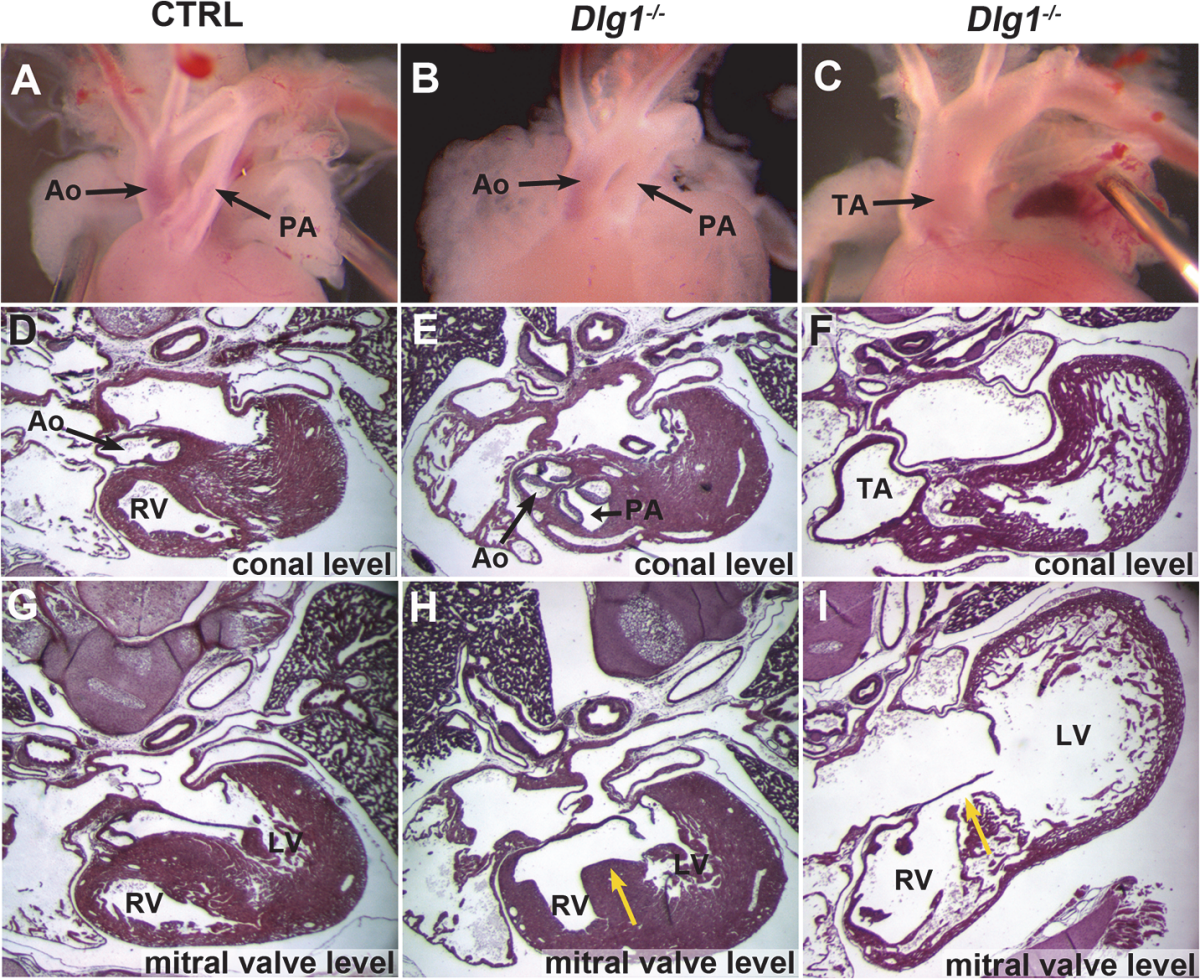
Cardiovascular defect in *Dlg1*-/- mice at E18.5. (A–C) Gross morphology of the outflow tract. Control mice (A) are normal, while *Dlg1* ^*-/-*^ mice exhibit parallel arteries (B) and PTA (C). (D–I) Paraffin sections indicating the inner structures of control (D, G) and two *Dlg1* ^*-/-*^ (E, H and F, I) mice. Panels D–F show sections at the conal level and panels G–I show sections of the same animals around the mitral valve. Pulmonary and aortic arteries connect to the right ventricle (E), indicating DORV. Truncus arteriosus is shown (F), indicating PTA. Opening of the ventricular septum in a *Dlg1* ^*-/-*^ mouse (yellow arrows in H and I) and the normal ventricular septum in a control mouse (G) are shown. Ao: aortic artery; LV: left ventricle; PA: pulmonary artery; RV: right ventricle; TA: truncus arteriosus.

**Fig 2:**
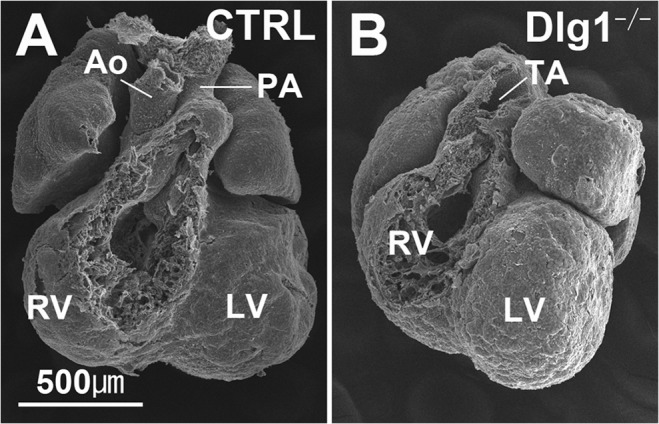
Scanning electron microscopy images of the heart and outflow tract at E12.5, The pulmonary artery connecting to the right ventricle and the aorta behind the pulmonary artery are distinguished in control mice (A), while they have not been separated in *Dlg1*-/- mice (B).

**Fig 3:**
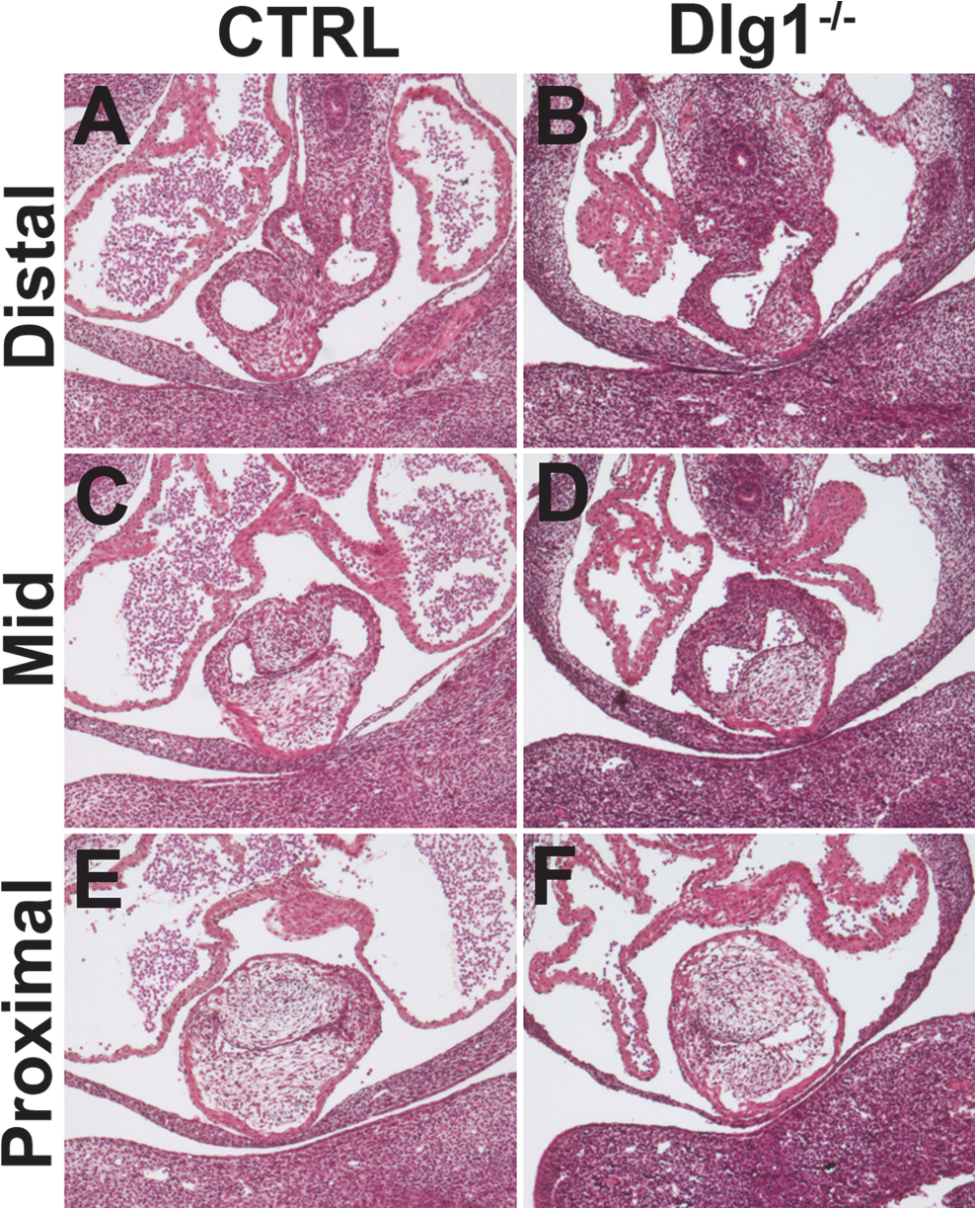
Disturbed septal formation in the outflow tract in *Dlg1*-/- mice at E11.5. The upper part of the outflow tract in the control mouse is completely separated into two distinct arteries (A), although fusion of the conotruncal cushions appears incomplete in the middle and proximal levels (C, D). Conotruncal cushions are formed in *Dlg1* ^*-/-*^ mice, but their fusion seems to be inhibited (B, D and F).

Null mutant mice for the *Dlg1* gene were reported to show postnatal lethality [26, 28, 30]. The mechanism for the lethality has not been elucidated. Because *Dlg1* ^*-/-*^ infant mice exhibit respiratory failure and cyanosis, we suspected a defect in the cardiovascular system and examined the gross anatomy of the heart outflow tract in *Dlg1* ^*-/-*^ mice at E18.5. In *Dlg1* ^*-/-*^ mice, malposition of the great arteries, in which the pulmonary artery and the aorta were parallel (Fig 1B) rather than crossing as they did in control mice (Fig 1A), and a single outflow tract indicating PTA (Fig 1C) were observed (Table 1). These abnormalities were not found in control mice. To further investigate the inner structure, serial paraffin sections were analyzed (Table 1). All *Dlg1* ^*-/-*^ mice examined exhibited ventricular septal defect (VSD) (Fig 1H and 1I; Table 1). Although some Dlg1^-/-^ embryos with the mixed background exhibited thin ventricular walls (Table 1; Fig 1F–1I), we did not further explore this finding in the present study. In addition, morphology indicating DORV (Fig 1E) or PTA (Fig 1F) was observed. Scanning electron microscope observation at E12.5 revealed that two arteries could be distinguished at the upper (distal to the heart) part of the outflow tract in all 10 control mice examined (Fig 2A), while the outflow tract was single in 4 of 5 *Dlg1* ^*-/-*^ embryos (Fig 2B). A similar defect was confirmed in serial paraffin sections at E11.5 (Fig 3). The distal part of the outflow tract was completely separated into two distinct arteries in 4 of 5 control mice examined (Fig 3A), while the outflow tract had a single continuous lumen in comparable sections in *Dlg1* ^*-/-*^ mice (Fig 3B).

**Table 1:** Summary of Dlg1 mutant cardiovascular phenotypes.

		C57BL/CBA mixed background		C57BL background	
		CTRL	Dlg1^-/-^	CTRL	Dlg1^-/-^
Gross morphology of outflow tract					
	Grossly normal	15 / 15	0 / 9	10 / 10	3 / 5
	Parallel	0 / 15	5 / 9	0 / 10	1 / 5
	PTA	0 / 15	4 / 9	0 / 10	1 / 5
Histological defects					
	VSD only	0 / 6	0 / 6	0 / 2	2 / 5
	VSD + DORV	0 / 6	2 / 6	0 / 2	2 / 5
	VSD + PTA	0 / 6	4 / 6	0 / 2	1 / 5
	ventricular wall thinning	0 / 6	2 / 6	0 / 2	0 / 5

**Fig 4:**
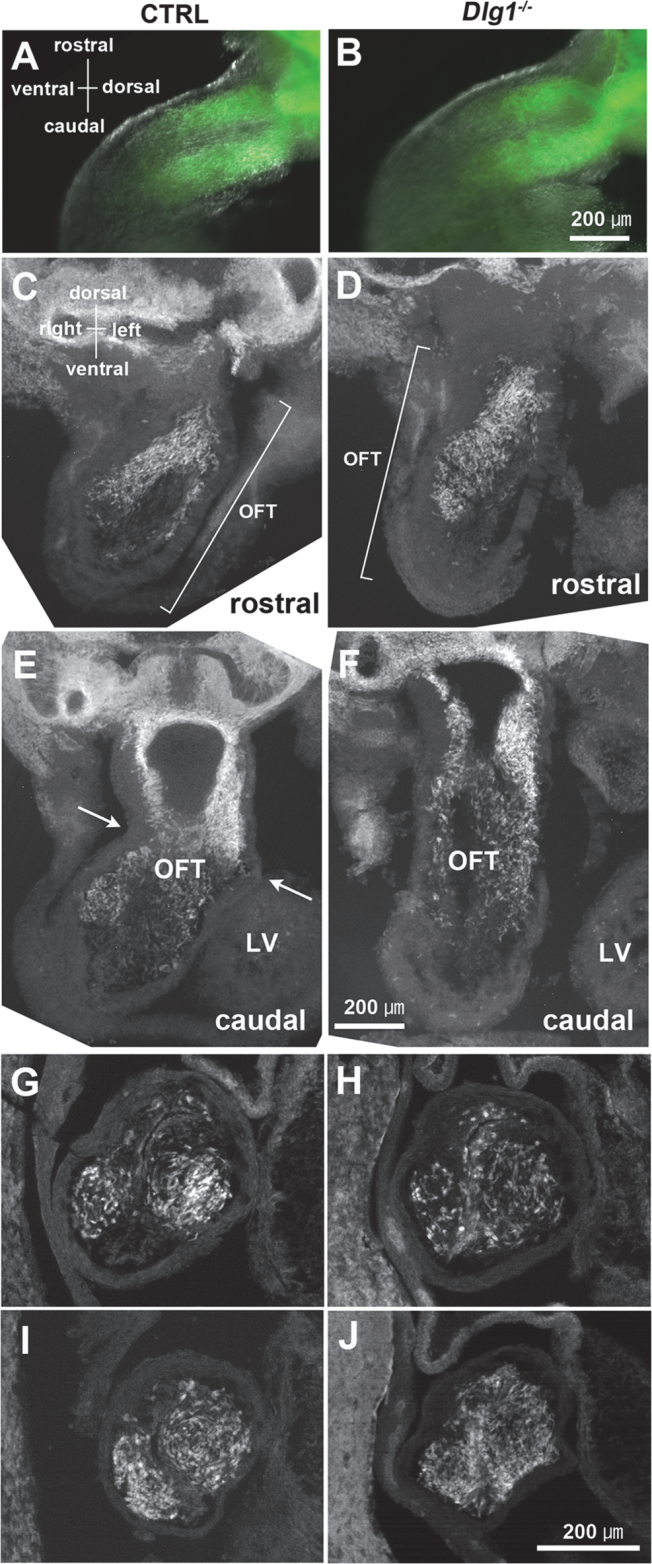
Outflow tract at E11.5 and distribution of neural crest cells. The left and right panels indicate control and *Dlg1* ^*-/-*^ mice, respectively. (A, B) Left-side view of the outflow tract in P0-Cre/CAG-EGFP transgenic mice. A bright-field microscopic image and an EGFP fluorescence image are merged. Neural crest-derived cells labeled by EGFP are migrating into the outflow tract as two discrete streams in control (A) and *Dlg1* ^*-/-*^ (B) mice. The left ventricle has been removed for photography, while the remnant is visible at the lower right region of panel B. (C–F) Fluorescent view of horizontal sections tangential to the outflow tract. Rostral (C, D) and caudal (E, F) sections from the same samples are shown. Sharp bending of the outflow tract is shown in the control mouse (arrows in E). (G–J) Cross-sections of the outflow tract in two control (G, I) and two *Dlg1* ^*-/-*^ (H, J) mice. The accumulation of neural crest cells within the conotruncal cushions sometimes seems irregular (H) or ectopic (J) in *Dlg1* ^*-/-*^ mice.

Cardiac neural crest cells migrate from the dorsal neural tube and form the aorticopulmonary septum and membranous portion of the ventricular septum. Therefore, we next examined whether the lack of *Dlg1* gene function affected the inflow of cardiac neural crest cells to the outflow tract using P0-Cre/EGFP transgenic mice, in which neural crest-derived cells were fluorescently-labeled with EGFP [36]. In our fluorescence stereomicroscopic observation, EGFP-positive cells were not detected in the outflow tract at E9.5, but were detected at E10.5 in *Dlg1* ^*+/+*^ mice expressing the P0-Cre/EGFP transgene (data not shown). Subsequently, we observed *Dlg1* ^*-/-*^ mice expressing the P0-Cre/EGFP transgene to examine the neural crest cell migration into the outflow tract at E11.5. Two streams of EGFP-positive cells were observed in the outflow tract in *Dlg1* ^*-/-*^ mice (Fig 4B and 4D–4F) as well as in control mice (Fig 4A and 4C–4E), and obvious retardation or disruption of the neural crest migration was not found. However, in cross-sections of the outflow tract, differences in the distribution pattern of neural crest cells were observed between genotypes (Fig 4G–4J). Neural crest cells were populated in outflow cushions with a tendency to condense at the center of the cushions in control mice (Fig 4G–4I). On the other hand, neural crest cells in *Dlg1* ^*-/-*^ mice were distributed rather evenly, or peripherally condensed at the comparable region of the outflow tract (Fig 4H–4J). Another feature of *Dlg1* ^*-/-*^ mice was identified during the observation of horizontal sections that were tangential to the outflow tract (Fig 4C–4F). In control mice, there was a sharp bend at the junction of the conus and the truncus (Fig 4E, arrows) [37], whereas the outflow tract in *Dlg1* ^*-/-*^ mice was straight (Fig 4F).

**Fig 5:**
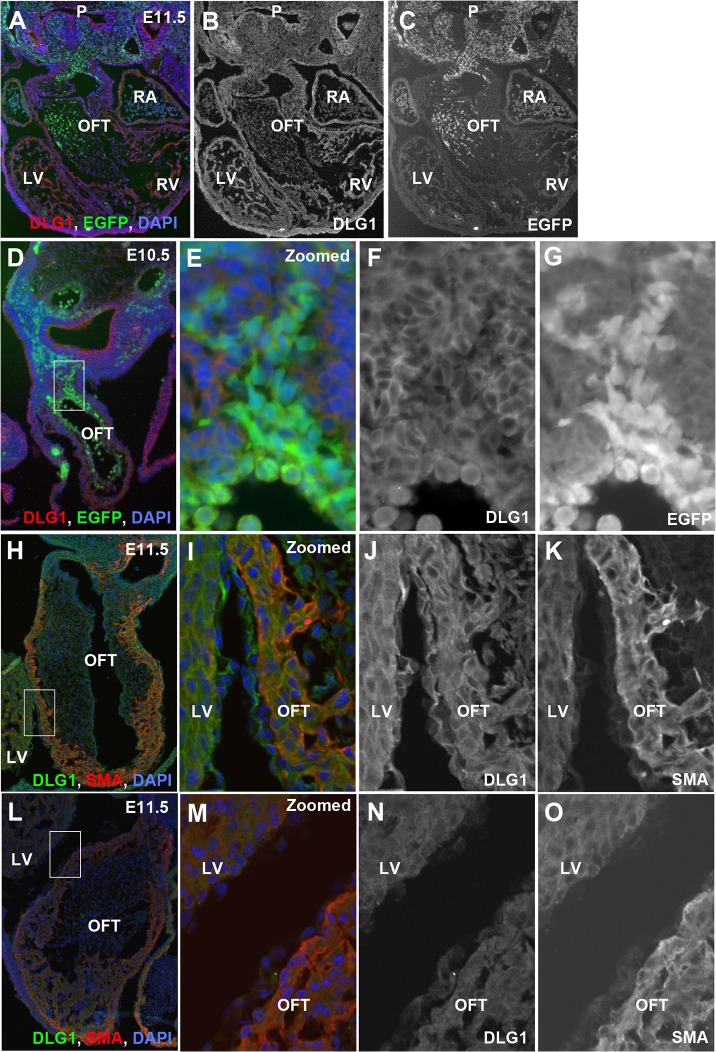
Expression of DLG1 during heart and outflow tract morphogenesis. (A–C) Expression of DLG1 protein (red in A and B) and distribution of neural crest-derived cells (green in A and C) in a P0-Cre/EGFP/*Dlg1* ^*+/+*^ mouse at E11.5. (D–G) Outflow tract in a P0-Cre/EGFP/*Dlg1* ^*+/+*^ mouse at E10.5 (D) and magnified images of the boxed area in panel D (E–G). The expression of DLG1 (red in D and E, and F) and distribution of neural crest-derived cells (green in D and E, and G) are shown. (H–O) Immunohistochemistry for DLG1 (green in H I, L, and M and J, N) and SMA (red in H, I, L, and M and K, O) in the outflow tract of *Dlg1* ^*+/+*^ (H–K) and *Dlg1* ^*-/-*^ (L–O) mice at E11.5. The whole outflow tract (H, L) and magnified images (I, M) corresponding to the boxed area are shown. LV: left ventricle; OFT: outflow tract; RA: auricle of right atrium; RV: right ventricle.

Next, we examined the expression of DLG1 protein in the developing heart region. DLG1 expression was detected in the ventricle, atrium, and outflow tract, although the signals were less prominent than those in the pharyngeal epithelial cell layer (Fig 5). The DLG1 signals were slightly weaker in EGFP-positive neural crest derived cells than in EGFP-negative cells (Fig 5D–5G) and relatively strong in the SMA-positive wall of the outflow tract (Fig 5H–5K). Fluorescent signals for DLG1 were found at cell-cell contact sites in *Dlg1* ^*+/+*^ mice (Fig 5F–5J), but were not detected in *Dlg1* ^*-/-*^ mice (Fig 5N).

### Shortening and extra hair cell rows of the organ of Corti

**Fig 6:**
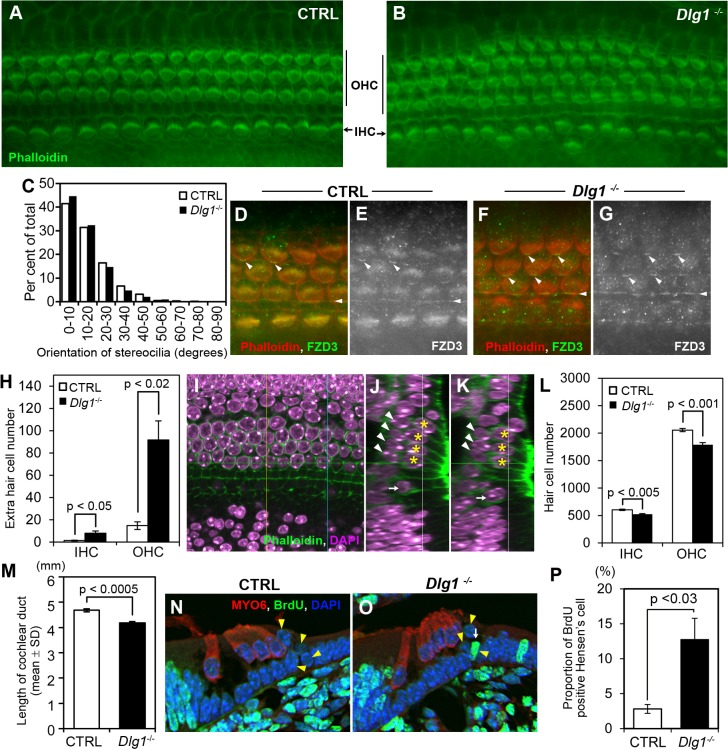
Analysis of the organ of Corti in *Dlg1* ^*-/-*^ mice at E18.5. (A, B) Surface preparation of the organ of Corti in control (A) and *Dlg1* ^*-/-*^ (B) mice stained with phalloidin (green). (C) Distribution of the stereocilia direction of the outer hair cells in the third row. The distribution of the stereocilia orientation of 100 hair cells was analyzed in 7 control and 6 *Dlg1* ^*-/-*^ mice. (D–G) Surface preparations of the organ of Corti in control (D, E) and *Dlg1* ^*-/-*^ (F, G) mice stained with phalloidin (red) and an anti-FZD3 antibody (green). (H) Total hair cell numbers in the extra row of inner hair cells (IHC) and outer hair cells (OHC) along an entire organ of Corti. (I–K) Alignment of hair cells and Deiters cells in *Dlg1* ^*-/-*^ mice at E18.5. (I) Optical section of a surface preparation stained with phalloidin (green) and DAPI (purple) at the vertical level of the white lines in panels J and K. (J, K) Z-plane images reconstructed by optical sections at the yellow line (J) and blue line (K) in panel I, respectively. The nuclei of the inner hair cells, outer hair cells, and Deiters cells are labeled with white arrows, yellow asterisks, and white arrowheads, respectively. (L) Total numbers of hair cells per organ of Corti. (M) Length of the cochlea. (N, O) Immunohistochemistry of cross-sections of the organ of Corti in control (N) and *Dlg1* ^*-/-*^ (O) mice at E18.5 that received BrdU injections between E14.5 and E16.5. Myosin 6 (red), BrdU (green), and nuclear (DAPI) staining is shown. Hair cells are labeled by myosin 6 staining. The yellow arrowheads and white arrow point to Hensen cells and a BrdU-positive Hensen cell, respectively. (P) Proportions of BrdU-positive cells in the Hensen cell populations.

Mutant mice deficient for PCP components such as WNT5A, FZDs, and VANGL2 were reported to have congenital heart defects [6, 15, 19]. Because the heart defects observed in *Dlg1* ^*-/-*^ mice were similar to those in PCP component-deficient mice, we examined whether DLG1 is necessary for PCP formation during mouse development. In mammals, hair cells of the organ of Corti reflect a defect in PCP signal transduction. Specifically, the orientation of the stereocilia, which are lined up in the same direction in normal mice, is disrupted when PCP signaling is defective [3]. Contrary to our expectation, the orientation of the stereocilia in the organ of Corti was not disrupted in *Dlg1* ^*-/-*^ mice (Fig 6A–6C). Furthermore, one of the PCP components, FZD3, showed a normal polarized distribution in *Dlg1* ^*-/-*^ mice as well as in control mice (Fig 6D–6G, arrowheads). Instead, *Dlg1* ^*-/-*^ mice exhibited an abnormal arrangement of hair cells in the organ of Corti. In contrast to control mice, in which one inner hair cell row and three outer hair cell rows were lined up along the cochlea, extra hair cell rows were apparent in *Dlg1* ^*-/-*^ mice (Fig 6B). The numbers of inner and outer hair cells in the extra hair cell rows were significantly higher in *Dlg1* ^*-/-*^ mice than in control mice (Fig 6H). Cochlear hair cells and Deiters cells, which are subjacent supporting cells, are differentiated from sensory epithelial cells [38]. Deiters cells have been reported to be missing in mice with impairment of the cell-fate-switching system [39]. To examine whether the cell-fate-regulating mechanism was disrupted, the possibility that Deiters cells were missing at the appearance of the extra rows of hair cells was examined by laser scanning confocal microscopy (Fig 6I–6K). In the area of extra hair cell rows where four outer hair cells were placed on a line (Fig 6J, yellow asterisks), Deiters cells (Fig 6J, white arrowheads) were increased in parallel to the hair cells, suggesting that the hair cells did not increase at the expense of the supporting cells. Conversely, the total numbers of hair cells were significantly decreased and the cochlear length was significantly shorter in *Dlg1* ^*-/-*^ mice than in control mice (Fig 6L and 6M). To further examine the possibility that hair cells were increased in *Dlg1* ^*-/-*^ mice, a BrdU labeling assay was performed. Cells in the organ of Corti undergo terminal proliferation by E14.5 and their proliferation is subsequently inhibited by the function of CDKN1B/p27kip1, such that proliferating cells are absent in the zone of non-proliferating cells thereafter [35]. The possibility that the hair cell proliferation arrest was released and the excessively generated hair cells caused extra rows was evaluated by detecting BrdU-positive cells in the zone of non-proliferating cells. In the regions of outer hair cells, pillar cells, and Deiters cells, no BrdU-positive cells were found in either control or *Dlg1* ^*-/-*^ mice. In the inner hair cell region, BrdU-positive cells were found in *Dlg1* ^*-/-*^ mice, but the incidence was not significantly high (0% in control mice vs. 0.37±0.37% in *Dlg1* ^*-/-*^ mice; p = 0.36). On the other hand, *Dlg1* ^*-/-*^ mice exhibited a significant increase in Hensen cells (Fig 6P), which appeared as tall columnar cells lying lateral to the outer hair cells (Fig 6N and 6O, yellow arrowheads). Taken together, these results exclude the possibility that an increase in hair cells caused the extra hair cell rows, and instead suggest that impaired tissue elongation during cochlear development led to the disarrangement of hair cells and extra hair cell rows.

### Defects in bone formation

**Fig 7:**
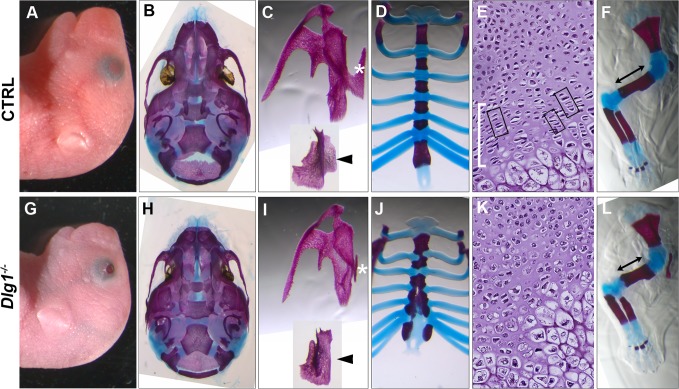
Skeletal defects in *Dlg1* ^*-/-*^ mice. Images of control (A–F) and *Dlg1* ^*-/-*^ (G–L) mice are shown. (A, G) Craniofacial morphology of the prenatal embryos at E18.5. *Dlg1* ^*-/-*^ mice invariably exhibit craniofacial hypoplasia and cyanosis, and often have open eyelids. (B, H) External surface of the cranial base. The mandibular bone has been removed. (C, I) Maxilla and palatal bone at E18.5. (D, J) Sternum at E18.5. (E, K) Hematoxylin and eosin-stained paraffin sections of the lower part of the sternum at E19.5. (F, L) Skeleton of the forelimb at E17.5.

Since *Dlg1* ^*-/-*^ mice have cleft palates and hypoplasia of the face [26] (Fig 7A–7G), we examined the craniofacial bone structure in *Dlg1* ^*-/-*^ mice. At E18.5, the facial bones were hypoplastic in *Dlg1* ^*-/-*^ mice (Fig 7B,7C,7H and 7I). The palatal process of the maxilla (Fig 7C–7I, asterisks) and the palatine process of the palatine bones (Fig 7C–7I, arrowheads) were contiguous at the midline in control mice (Fig 7B and 7C), but were invariably hypoplastic with absence of the palatal shelf in *Dlg1* ^*-/-*^ littermates (Fig 7H and 7I). This hypoplasia of the craniofacial bones is a common phenotype in mice with a neural crest defect [40] and suggests some effects of DLG1 deficiency on the behavior of cranial neural crest cells.

Other than the craniofacial region, we noticed malformation of the sternum in *Dlg1* ^*-/-*^ embryos during the course of our observation of the cardiovascular system. In control mice, the sternal bars had completely fused at the midline by E18.5 and cartilaginous tissues at the level of the rib head and mineralized tissues were regularly banded (Fig 7D). However, in *Dlg1* ^*-/-*^ mice, the fusion of the sternal bars had not been completed in the lower sternum at E18.5 (Fig 7J). The length of the sternum in *Dlg1* ^*-/-*^ mice (2.78±0.12 mm) was equivalent to that in control mice (2.99±0.18 mm) at E15.5 (p = 0.53), but significantly shorter than that in control mice at E16.5 (3.23±0.07 mm vs. 4.04±0.23 mm; p<0.03), E17.5 (4.13±0.17 mm vs. 5.17±0.08 mm; p<0.0001), and E18.5 (5.17±0.15 mm vs. 6.30±0.18 mm; p<0.0003). In addition, the mineralization pattern was irregular and the sternal tissue appeared calcified without sufficient longitudinal elongation of the sternum (Fig 7J). In the observation of the cartilaginous tissue of the sternum at E19.5, the chondrocytes were stacked in columns (Fig 7E, boxes) within the zone of proliferation (Fig 7E, white bar) in control mice, while the columnar structure was disorganized in *Dlg1* ^*-/-*^ mice (Fig 7K). In addition, the long bones of the limbs were shorter in *Dlg1* ^*-/-*^ mice. In the upper limbs, for example, the mean lengths of the humerus were 4.37±0.040 mm and 3.57±0.074 mm in control and *Dlg1* ^*-/-*^ mice at E18.5, respectively, with a significant difference (p<0.0001; Fig 7F–7L, arrows). The long bone diameters were evaluated by measuring the width of the humerus at the narrowest point in the photograph. The widths were 0.513±0.0073 mm and 0.591±0.0051 mm in control and *Dlg1* ^*-/-*^ mice, respectively, and the difference was significant (p<0.0001). These results indicate defective cartilaginous cell arrangement and tissue elongation during bone development in *Dlg1* ^*-/-*^ mice.

### Shortening of the intestine

**Fig 8:**
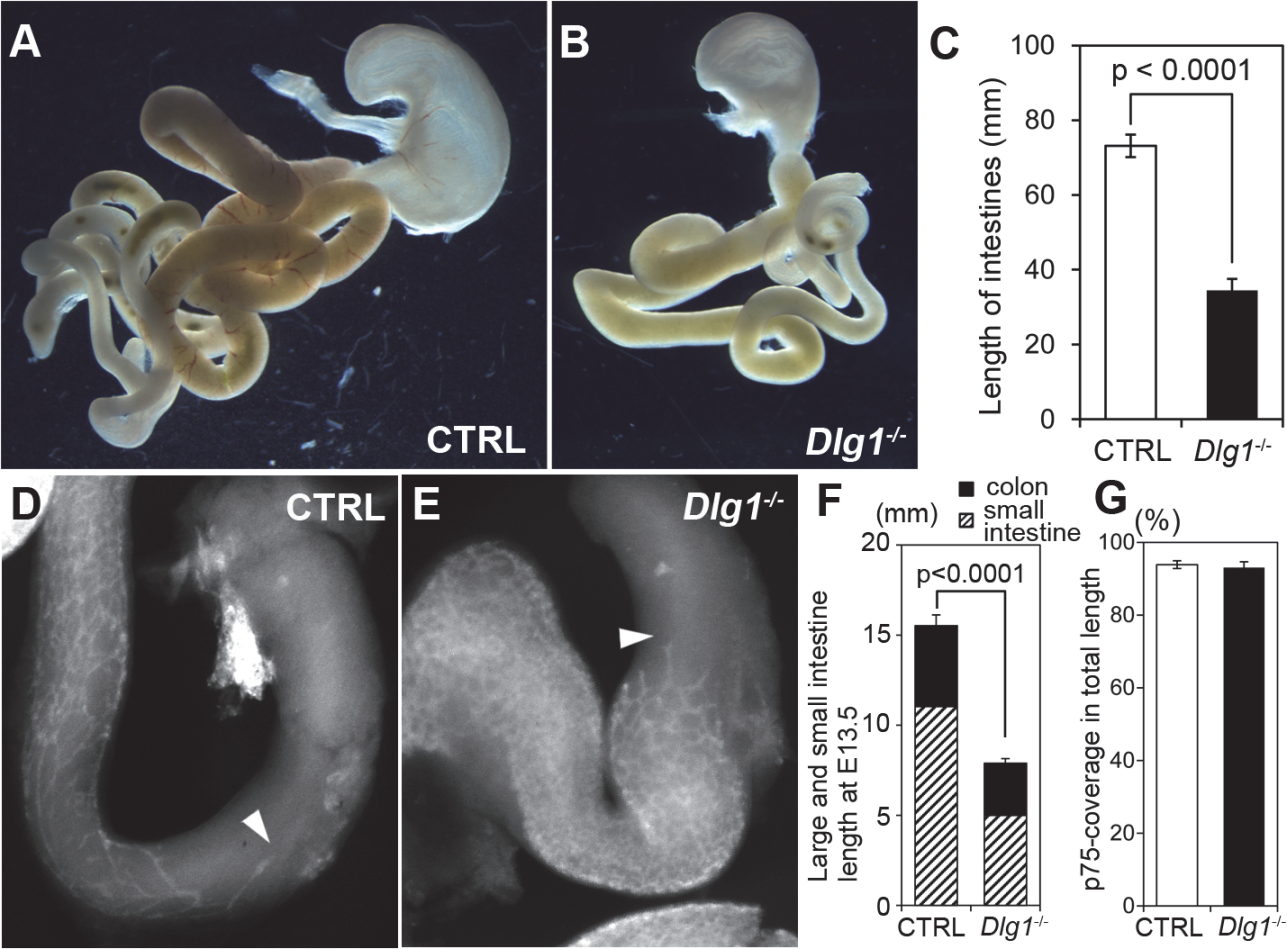
Shortening of the intestine in *Dlg1* ^*-/-*^ mice. (A, B) Digestive tract at E18.5. The digestive tract in the *Dlg1* ^*-/-*^ mouse (B) is smaller than that in the control mouse (A). (C) Length of the intestine at E18.5. (D, E) Whole-mount immunohistochemistry for p75 protein in control (D) and *Dlg1* ^*-/-*^ (E) mice at E13.5. (F) Intestinal length of each segment at E13.5. The intestinal length is significantly shorter in *Dlg1* ^*-/-*^ mice than in control mice. The ratios of the small and large intestine are unchanged in *Dlg1* ^*-/-*^ mice. (G) Coverage proportion of the p75-positive enteric nerve plexus in the whole length of the intestine at E13.5. There is no significant difference between the genotypes.

To determine whether the loss of DLG1 function affects the tissue elongation in other organs, we examined the length of the intestine, whose elongation is dependent on WNT5A, a ligand in the PCP pathway [41]. The length of the intestine was remarkably short in *Dlg1* ^*-/-*^ embryos at E18.5 (Fig 8A–8C), indicating that DLG1 is involved in the elongation process during gut development. For completion of the functional development of the gut, formation of the enteric nerve plexus is crucial as well as endodermal intestinal tube formation. The enteric nerve plexus covers the surface of the tract and regulates intestinal peristalsis. The enteric nerve plexus is composed of neural crest cells migrating downward along the intestine [40]. Defective migration of neural crest cells and insufficient coverage of the intestinal surface by the nerve plexus result in Hirschsprung’s disease in humans with severe malfunction of digestion. Since several neural crest cell-derived tissues and organs, such as the craniofacial skeleton and septum of the heart outflow tract, exhibited malformations in *Dlg1* ^*-/-*^ mice, the development of the enteric nerve plexus was examined. The distribution of the enteric nerve plexus was observed at E13.5, when the neural crest cells were covering the intestine. The enteric nerve plexus was visualized by whole-mount immunohistochemistry for NGFR/p75 protein and the coverage along the intestinal surface was compared between the genotypes (Fig 8D–8G). At this developmental stage, the overall length of the intestine in *Dlg1* ^*-/-*^ mice was already significantly shorter than that in control mice (Fig 8F), while the percentages of plexus coverage over the whole length of the intestine were equivalent between control and *Dlg1* ^*-/-*^ mice, showing no significant difference (Fig 8G). Taken together, DLG1 deficiency seems to severely impair the elongation process of the entire length of the small and large intestine, but does not affect the behavior of enteric neural crest-derived cells.

## Discussion

### Novel cardiovascular phenotypes in *Dlg1* ^*-/-*^ mice

In our previous reports, we described abnormal development of the urogenital organs in *Dlg1* ^*-/-*^ mice [28, 42], but the cause of the neonatal lethality and the cellular mechanisms underlying the developmental defects in these mice remained unclear. In the present study, we further investigated the phenotypes of *Dlg1* ^*-/-*^ mice to identify the cause of the lethality and to gain clues toward elucidating the mechanism for DLG1 function. As a result, we found severe morphological abnormalities in the cardiovascular system, which have not previously been reported. These defects can cause abnormal blood flow and the cyanosis and perinatal death observed in *Dlg1* ^*-/-*^ mice. The phenotypes of *Dlg1* ^*-/-*^ mice exhibited variation. Since VSD was observed in all *Dlg1* ^*-/-*^ mice, the process for alignment and fusion of conotruncal and endocardial cushions seemed to be affected in *Dlg1* ^*-/-*^ mice. In severe cases, additional malformation of the outflow tract such as PTA or DORV was found. These phenotypes are known to be caused by abnormal development of cardiac neural crest cells and/or cells derived from the SHF [43, 44]. The present results showed abnormal assembly patterns of cardiac neural crest cells in the outflow tract in *Dlg1* ^*-/-*^ mice. In addition, the straight outflow tract in *Dlg1* ^*-/-*^ mice suggests deficient deployment of SHF-derived cells into the outflow tract [37]. In control mice, DLG1 expression in the outflow tract was detected in both SMA-positive cells and EGFP-positive cells, which are considered to be derived from the SHF and neural crest, respectively. Therefore, it remains to be determined in future studies which cell type is responsible for the outflow defects in *Dlg1* ^*-/-*^ mice.

### PCP phenotypes in *Dlg1* ^*-/-*^ mice

Cells in multicellular organisms establish vertical apicobasal polarity and horizontal PCP. During PCP formation in *Drosophila* for example, the PCP signaling cascade, including the core PCP components of DSH, FZ, VANG, STAN, PK, and DGO, plays a central role. In addition, many other effectors and modifiers of this signaling cascade have been reported [45]. In vertebrates, the PCP signaling pathway is one of the non-canonical WNT pathways and is activated by binding of the WNT ligand to FZD receptors [46]. SCRIB, which is important for apicobasal polarization, is also involved in PCP regulation [3]. PCP signals exert their roles during animal development in two aspects: first, in planar cell polarization in its narrow sense, in which each cell orients itself to the correct direction in a plane; and second, in CE, in which the tissue extends its length by narrowing its width by cell rearrangement through cell intercalation or reconstruction of cell-cell junctions [5, 47]. Mutant mouse strains deficient for PCP component genes, including *Fzd1* and *Fzd2* double-mutant mice, looptail mice with a *Vangl2* gene mutation, and circletail mice with a *Scrib* gene mutation, exhibit cleft palates and defects in ventricular septation and outflow tract alignment [15, 16, 19, 48]. As the phenotypes of *Dlg1* ^*-/-*^ mice in the palate and cardiovascular system were very similar to those of PCP-deficient mice, we intended to evaluate the possibility that DLG1 functions in the PCP signaling cascade. In mammals, regulation of the orientation of the stereocilia in hair cells and lengthening of the organ of Corti are known to require PCP signaling [3, 4]. Actually, in the present study, cochlear shortening was observed in *Dlg1* ^*-/-*^ mice, while misorientation of the stereocilia was not detected. These results raise the possibility that DLG1 is involved in PCP signaling.

### Possible CE impairment in *Dlg1* ^*-/-*^ mice

CE and planar cell orientation are both affected by deficiency of PCP genes. Although the mechanisms for regulating cell orientation and CE under PCP signaling remain unclear, CTNND1 (p120-catenin), which exerts its role in cell-cell adhesion through recruitment of cadherins to the cell surface, was recently reported to be required for CE, but not for hair cell orientation in cochlear development [49]. This indicates that both CE and planar cell polarization require PCP signaling, but each process is regulated by distinct mechanisms in the downstream. The present results showing a severe disruption of tissue elongation and undamaged PCP in *Dlg1* ^*-/-*^ mice may suggest a role for DLG1 in the regulation of CE. Our present and previous findings that the elongations of the ureter, intestine, and long bones, which require PCP signaling and are considered to depend on CE, were impaired in *Dlg1* ^*-/-*^ mice support this idea [28, 50, 51].

The relationship between DLG1 and PCP signaling is presently unknown. However, DLG1 may contribute to the cellular responses downstream of PCP signaling, because our results showed that DLG1 deficiency did not affect the localized distribution of FZD3. Future studies are required to evaluate this possibility.

### Developmental aspects of the organ of Corti other than CE

The appearance of extra rows of hair cells in the organ of Corti can be caused by mechanisms other than impaired CE. One possible mechanism is that an increase in hair cells by cell cycle dysregulation caused the appearance of the extra row. Deficiency of the cell cycle-regulating protein CDKN1B/p27kip1 has been reported to be the cause [35]. Another possible mechanism is impairment of the cell fate determination mechanism regulated by Notch signaling [4, 52]. In Notch signaling-deficient mice, appearance of extra hair cell rows by increased differentiation into hair cells and compensatory missing of supporting cells have been reported [53]. In the case of *Dlg1* ^*-/-*^ mice, however, the total number of hair cells was decreased and supporting cells beneath the extra hair cell row were not missing. Therefore, Notch signaling and CDKN1B/p27kip1 function do not seem to be related to the appearance of the extra hair cell rows. Nonetheless, there does appear to be some problem associated with the mechanism that arrests the cell proliferation in *Dlg1* ^*-/-*^ mice, as more Hensen cells underwent delayed proliferation in these mice. The significance of this alteration for organogenesis needs to be evaluated in future studies.

### Different phenotypes among mouse strains

During the course of the present investigation, Rivera et al. [54] studied two *Dlg1* gene-null mutant mouse strains with a C57BL/6J or FVB/NJ background and expressed the idea that DLG1 is involved in PCP. The present results, which were mostly obtained using a *Dlg1* gene-null mutant mouse strain with its genome derived from the C57BL/6N and CBA/J mouse strains, also indicate a role for DLG1 in PCP phenomena, generally supporting the opinion of Rivera et al. However, the phenotypes described in Rivera et al. and our present report consist of not only universal phenotypes that were observed in all three mouse strains, but also specific phenotypes with varied severity or incidence depending on the different mouse strains. Among the phenotypes reported by Rivera et al., lethality, cleft palate, short snout, and reduced size seemed to be universal phenotypes as they were observed in all three mouse strains, although we did not mention all of these phenotypes in this paper. On the other hand, among the comparable phenotypes, the hair cell misorientation was specific for the C57BL/6J strain in Rivera et al., and the long bone shortening was specific for the FVB/NL strain in Rivera et al. and our strain. Since the universal phenotypes represent phenomena in which DLG1 plays a central role with little interference from other genetic backgrounds, their identification will be important to fully evaluate the role of DLG1. The cardiovascular defect and disruption of tissue extension in the intestine and organ of Corti found in the present study were not mentioned in the report by Rivera et al. It would be of interest to clarify whether these phenotypes are observed in *Dlg1* ^*-/-*^ mice with a C57BL/6N or FVB background.

## Conclusions

In the present study, we observed the importance of DLG1 for the first time in cardiovascular development, which would explain the neonatal lethality of *Dlg1* ^*-/-*^ mice. In addition, we showed that DLG1 is involved in the tissue elongation of the cochlea, gut, and bone, suggesting the involvement of DLG1 in PCP processes, particularly in CE movement. Further investigation of DLG1-dependent organogenesis can be expected to clarify the contribution of CE to each developmental process and help toward understanding the mechanism of DLG1 function.
